# Postoperative Debridement versus No Debridement in Chronic Rhinosinusitis

**DOI:** 10.22038/ijorl.2020.43210.2417

**Published:** 2021-01

**Authors:** Sara Rahavi-Ezabadi, Amin Amali, Babak Saedi, Nafiseh Gilanifar, Fatemeh Mirashrafi

**Affiliations:** 1 *Otorhinolaryngology Research Center, Department of Otorhinolaryngology Head and Neck Surgery, Imam Khomeini Hospital Complex, Tehran University of Medical Sciences, Tehran, Iran.*

**Keywords:** Chronic sinusitis, Debridement, Endoscopy, Post-operative care

## Abstract

**Introduction:**

This study aimed to assess the long-term effects of postoperative debridement on the 4^th^ and 8^th^ postoperative weeks versus no debridement in terms of subjective and objective outcomes.

**Materials and Methods:**

The statistical population of this study (n=80) consisted of 40 patients having chronic rhinosinusitis with nasal polyposis (CRSwNP) and 40 patients having chronic rhinosinusitis without nasal polyposis (CRSsNP). These samples were randomly divided into two groups of debridement and control.

**Results:**

According to the results, 8 weeks after endoscopic sinus surgery (ESS), the 22-item Sino-Nasal Outcome Test questionnaire score (P =0.03), Lund-Kennedy score (P<0.001), nasal blockage (P=0.02), and loss/decrease in sense of smell (P=0.02) in CRSwNP were significantly lower in the debridement group than in the control group. Moreover, 6 months after ESS, in both CRSwNP and CRSsNP, no significant difference was observed between the two groups considering the outcomes (P>0.05).

**Conclusion:**

This study showed that debridement could lead to short-term improvements in CRSwNP patients; however, no long-term benefit was observed.

## Introduction

Chronic rhinosinusitis (CRS) is a common chronic disease with a significant impact on patients and healthcare systems (1). Patients with CRS have nasal blockage, anterior or posterior nasal discharge, facial pain or pressure, and reduced or absent sense of smell that lasts more than 12 weeks with objective endoscopic or imaging findings (2). This condition is clinically classified into two groups, namely CRS with nasal polyposis (CRSwNP) and CRS without nasal polyposis (CRSsNP). These two subsets share some common clinical features; however, they often differ on the molecular level, management, and prognosis (3). 

Pharmacological therapy is considered the first treatment of CRS and endoscopic sinus surgery (ESS) is reserved for medically resisted CRS. The aim of ESS is to clear inflammation and infection, re-establish physiological sinus ventilation and drainage, and allow delivery of topical medications (4,5). 

Postoperative care, including nasal irrigation and topical steroid, has been suggested to improve patient outcomes (6,7). There is a controversy in the literature regarding the efficacy, timing, and frequency of debridement in the postoperative period. Postoperative debridement helps to remove the crusts, clots, and secretions, which are believed to serve as a framework for scar development.

A body of evidence suggests that postoperative debridement decreases the risk of adhesions at 3 months follow-up; nonetheless, the impact on longer-term outcome is not clear (8,9). Although the adoption of such a procedure has some advantages in decreasing synechia, there are several difficulties in performing it. Debridement can increase postoperative pain and cause discomfort. Frequent postoperative visits are time-consuming, expensive (10). Since it is not clear whether the advantages of this procedure outweigh its difficulties or not, rhinologists adopt a wide range of measures to determine the optimal number and timing for debridement after ESS. 

This study was conducted to assess the long-term effects of postoperative debridement on the 4^th^ and 8^th^ postoperative weeks versus no debridement in terms of subjective quality of life scores, symptoms scores, and objective endoscopic scores. This study was the first randomized control trial (RCT) that compare the effects of debridement in CRSwNP with CRSsNP patients.

## Materials and Methods

**Study population**

This study was designed as a randomized unicenter trial to investigate the subjective and objective benefits and drawbacks of postoperative debridement versus no debridement in CRSwNP and CRSsNP parallel groups. The present study has been registered by the Iranian Registry of Clinical Trials. Full ethical approval was granted by the Research Ethics Committee of Tehran University of Medical Sciences, Tehran, Iran (201521240). The population of this study consisted of patients with CRSwNP and patients with CRSsNP (n=40 each) who attended the department of Otorhinolaryngology of Valiasr hospital within February 2016-January 2017. The purpose of the study was explained to the patients and informed consent was obtained from them. They were also informed about the possibility of study withdrawal at any research stage without concerning about further treatment. Medically resistant CRS patients with and without nasal polyposis who were candidates for primary sinus surgery were entered into the study. The exclusion criteria included being under 18 years, having previous sinus surgery, cystic fibrosis, congenital mucociliary problems, immune deficiency, systemic vasculitis, severe septal deviation, craniofacial malformation, allergic fungal rhinosinusitis, and fungal rhinosinusitis. Smokers were asked to stop smoking 3 to 4 weeks before the surgery. Patients who did not quit smoking were excluded from the study.

**Randomization**

Both CRSwNP (n=40) and CRSsNP (n=40) patients were enrolled in the study and registered by contacting the clinical trial coordinator. Randomization took place in the operating room after ESS, by opening a sealed, numbered envelope. 

The envelopes were prepared by the trial statistician using the block randomization method (block size of 4).The debridement was performed in an outpatient clinic on the 4^th^ and 8^th^ weeks postoperatively, during which clots, crusts, and secretions were evacuated by suction or other gentle instruments. However, fixed clots and crusts were left untouched. Patients, endoscopists, and investigators who analyzed the outcomes were unaware of the randomization schedule.

**Clinical evaluation**

After completing a full history and physical examination, baseline clinical data were gathered. Any history of allergy, asthma, or aspirin hypersensitivity was recorded. Patients with allergy symptoms or a positive history were recommended to undergo allergy testing for confirmation. The endoscopic staging was based on the Lund-Kennedy endoscopy scoring system (11). A computed tomography (CT) scan of the paranasal sinuses was scored using the Lund-Mackay scoring system (12). The patient's symptoms were assessed using a visual analogue scale (VAS) for five major symptoms, including 1) nasal blockage, 2) anterior or posterior nasal discharge, 3) facial pain or pressure, 4) headache, and 5) reduction or loss of smell. Health-related quality of life was measured using the 22-item Sino-Nasal Outcome Test (SNOT-22) questionnaire (13).

All patients under general anaesthesia underwent full ESS using the same technique, including antrostomy, ethmoidectomy, sphenoidotomy, and (if necessary) frontal recess approach. All patients received preoperative oral steroid for 10 days (Prednisolon, 20 mg/ day). The surgery was bilateral in all patients. All patients used saline serum nasal irrigation three times daily after surgery and nasal budesonide spray daily from day 7 postoperatively. 

**Follow up**

All patients were monitored for adverse events at 4 and 8 weeks and 6 months postoperatively. In the intervention group, the debridement was performed by the endoscopist who was blinded to the study, on the 4^th ^and 8^th^ weeks. Lund-Kennedy, SNOT-22, and VAS scores were documented again at the 8-week and 6-month visits.

**Statistical analysis**

This study would have 80% power to detect the difference between two groups with n=18 per group. This calculation was based on a two-sample t-test with a two-sided alternative hypothesis that assumed equal group variances and a type one error level of 5%. The sample size was increased to 20 per group assuming a potential 10% loss to follow-up rate. All collected data were analyzed in SPSS software (version 15). 

The distribution of continuous variables was analyzed using the Kolmogorov-Smirnov test for normality. Baseline variables were compared between the two groups using the independent Student’s t-test, Mann–Whitney U, and Chi-square tests. Nominal variables were tested using the x^2^ test. A p-value of less than 0.05 was considered statistically significant. 

## Results

In this study, 40 patients with CRSwNP and 40 patients with CRSsNP participated who fulfilled the inclusion criteria. The patients were followed for 6 months. During the research Process, 4 patients with CRSwNP and 5 patients with CRSsNP were lost to follow-up at 6 months. The patients' demographic information is summarized in Table 1. 

At the baseline, no significant difference was identified between the debridement and control group in CRSwNP or CRSsNP patients in terms of age, gender, smoking, allergy, asthma, CT scan score, and frontal sinus involvement. There was also no significant difference between debridement and control group regarding disease severity as measured by VAS, SNOT-22 score, and Lund-Kennedy endoscopic score systems (Table.1).

**8 weeks after endoscopic sinus surgery**

The SNOT-22 score obtained for CRSwNP was significantly lower in the debridement group than that in the control group (13.08±15.17 vs. 19.4±9.89, P-value=0.03). Regarding CRSsNP, although the score in the debridement group was higher than that in the control group, it was not significant (17.7±9.5 vs. 12.4±9.1, P-value=0.09; Table.2).

**Table 1:** Demographic information of patients having chronic rhinosinusitis with and without nasal polyposis

	Patients having chronic rhinosinusitis with nasal polyposis			Patients having chronic rhinosinusitis without nasal polyposis		
Characteristic	Debridement n=20	No debridement n=20	P-value	Debridement n=20	No debridement n=20	P-value
Age, Mean (SD)	37.8 (11.3)	41.1 (11.9)	0.36	40.1	36.9	0.38
Male gender, n (%)	12 (60)	10 (50)	0.52	11 (55)	13 (65)	0.51
Asthma, n (%)	5 (25)	4 (20)	0.70	4 (20)	2 (10)	0.37
Allergy, n (%)	6 (30)	7 (35)	0.73	4 (20)	5 (25)	0.70
Smoking, n (%) Pack year, Mean (SD)	4 (20) 3.5 (4.8)	5 (25) 3.3 (5.3)	0.70 0.88	6 (30) 3.4 (5.48)	4 (20) 2.9 (5.15)	0.46 0.74
Lund-Mackay score (SD)	17.5 (4.8)	18.8 (4.5)	0.42	14.8 (4.3)	14.1 (4.7)	0.62
Lund-Kennedy score (SD)	9.1 (2.3)	9.6 (2.1)	0.48	6.7 (1.6)	6.4 (1.5)	0.54
22-item Sino-Nasal Outcome Test questionnaire score	38.4 (15.1)	39.5 (15.9)	0.82	32.6 (14.2)	34.5 (13.8)	0.67

**Table 2:** Obtained scores of 22-item Sino-Nasal Outcome Test questionnaire

	Patients having chronic rhinosinusitis with nasal polyposis			Patients having chronic rhinosinusitis without nasal polyposis		
	Debridement	No debridement	P-value	Debridement	No debridement	P-value
SNOT-22 Score before treatment, Mean (SD)	38.4 (15.1)	39.5 (15.9)	0.82	32.6 (14.2)	34.5 (13.8)	0.67
SNOT-22 score at 8 weeks, Mean (SD)	13.0 (8.5)	19.4 (9.8)	0.03	17.7 (9.5)	12.4 (9.1)	0.09
SNOT-22 score at 6 months, Mean (SD)	14.6 (8.4)	16.3 (9.1)	0.56	10.1 (8.2)	8.4 (7.8)	0.53

SNOT-22: 22-item Sino-Nasal Outcome Test

Nasal blockage and loss/decrease in sense of smell, in CRSwNP, were both lower in the debridement group compared to those in the control group (nasal blockage: 1.4±1.25 vs. 2.4±1.42, P-value=0.02; loss/decrease in sense of smell: 0.6±0.81 vs. 1.3±1.02, P-value=0.02). In CRSsNP, no significant difference was observed between the debridement and control groups regarding nasal blockage and loss/decrease in sense of smell (1.4±1.59 vs. 2.1±1.48, P-value=0.17; 0.75±0.97 vs. 1.2±1.13, P-value=0.20). Both in CRSwNP and CRSsNP, there was no significant difference between debridement and control groups in terms of nasal discharge, facial pain, and headache (Table.3).

The Lund-Kennedy score, in CRSwNP, was significantly lower in the debridement group than in the control group (5.1±1.9 vs. 8.4±2.01, P<0.001). In CRSsNP, there was no statistically significant difference between the debridement and control groups on the Lund-Kennedy score (4.02±1.55 vs. 4.7±1.64, P- =0.20; Table.4).

**Table 3:** Symptoms scores

		**Patients having chronic rhinosinusitis with nasal polyposis **			**Patients having chronic rhinosinusitis without nasal polyposis**		
		**Debridement**	**No debridement**	**P-value**	**Debridement**	**No debridement**	**P-value**
Nasal blockage, VAS score, Mean (SD)	Before treatment	7.6 (2.8)	7.8 (2.9)	0.82	6.8 (2.4)	6.5 (2.2)	0.68
	at 8 weeks	1.4 (1.2)	2.4 (1.4)	0.02	1.4 (1.6)	2.1 (1.4)	0.17
	at 6 months	2.5 (1.4)	2.8 (1.5)	0.55	1.7 (1.3)	1.9 (1.3)	0.66
Nasal discharge, VAS score, Mean (SD)	Before treatment	7.3 (2.7)	7.4 (2.8)	0.91	7.2 (2.6)	7.0 (2.7)	0.81
	at 8 weeks	2.3 (1.4)	3.1 (1.6)	0.12	2.0 (1.3)	1.8 (1.5)	0.75
	at 6 months	2.0 (1.2)	2.3 (1.3)	0.49	1.9 (1.4)	1.8 (1.5)	0.84
Loss of smell, VAS score, Mean (SD)	Before treatment	6.8 (2.2)	6.7 (2.5)	0.89	5.9 (2.3)	6.1 (2.4)	0.79
	at 8 weeks	0.6 (0.8)	1.3 (1.0)	0.02	0.7 (0.9)	1.2 (1.1)	0.20
	at 6 months	1.1 (1.2)	1.5 (1.2)	0.34	0.9 (1.1)	0.9 (0.9)	0.91
Facial pain, VAS score, Mean (SD)	Before treatment	5.9 (2.2)	5.7 (2.2)	0.78	5.5 (2.0)	5.9 (2.6)	0.49
	at 8 weeks	1.5 (1.1)	1.7 (1.3)	0.61	1.3 (1.2)	1.5 (1.0)	0.61
	at 6 months	2.1 (1.4)	2.3(1.3)	0.67	1.4 (1.2)	1.3 (1.3)	0.82
Headache, VAS score, Mean (SD)	Before treatment	4.5 (1.9)	4.3 (2.1)	0.75	4.4 (2.1)	4.3 (1.7)	0.93
	at 8 weeks	1.9 (1.5)	2.3 (1.6)	0.43	2.3 (1.6)	2.0 (1.6)	0.65
	at 6 months	2 (1.4)	1.8 (1.2)	0.65	1.9 (1.5)	1.8 (1.3)	0.81

VAS: visual analogue scale

**Table 4:** Lund Kennedy Scores

	**Patients having chronic rhinosinusitis with nasal polyposis **			**Patients having chronic rhinosinusitis without nasal polyposis**		
	**Debridement**	**No debridement**	**P-value**	**Debridement**	**No debridement**	**P-value**
Lund-Kennedy Score before treatment, Mean (SD)	9.1 (2.3)	9.6 (2.1)	0.48	6.7 (1.6)	6.4 (1.5)	0.54
Lund-Kennedy score at 8 weeks, Mean (SD)	5.1 (1.9)	8.4 (2.0)	<0.001	4.0 (1.5)	4.7 (1.6)	0.20
Lund-Kennedy score at 6 months, Mean (SD)	3.4 (1.6)	4.2 (1.3)	0.11	2.8 (1.7)	3.2 (1.3)	0.45

**6 months after endoscopic sinus surgery**

Both in CRSwNP and CRSsNP, there was no significant difference between debridement and control groups on the SNOT-22 score.

In CRSwNP and CRSsNP, the debridement group showed a lower Lund-Kennedy score than the control group; however, it was not significant. No significant differences were observed between the two groups in CRSwNP and CRSsNP patients in terms of nasal blockage, nasal discharge, loss/decrease in sense of smell, facial pain, and headache. Lund-Kennedy or SNOT-22 scores showed no significant correlation with allergy, asthma, ASA sensitivity, and smoking in both groups. 

**Adverse event**

All patients underwent follow-up visits for 6 months, during which no major complication was reported. Two patients had minor epistaxis after ESS which was responsive to conservative management.

## Discussion

The role of nasal debridement in improving postoperative outcomes is controversial. Periodic postoperative debridement helps to remove blood clots, crusts, and secretions leading to the provision of better nasal breathing and delivery of nasal steroids. Crusts and clots may trap mucus and serve as a culture medium for pathogens. They also may act as an inflammatory bridge and lead to scar formation (14). Despite having some advantages, postoperative debridement may cause inconvenience, pain, and bleeding. It is time-consuming and increases the cost of caring since more frequent postoperative visits are required (10). The results of a systematic review performed by Tzelnick, including 4 studies (152 patients), revealed that the quality of life and disease severity scores were not significantly different between debridement and control groups. A lower risk of adhesion at 3 months follow-up was found in the debridement group; however, there was a small body of evidence to make such a conclusion (8). Fishman et al. found that no significant difference in endoscopic appearance or symptom scores was seen between debridement and control groups. Subgroup analysis showed a significant effect of debridement on adhesion at 3 months but not on oedema, polyp, granulation, discharge, or crusting (9). 

In a literature review conducted by Green et al., 6 RCTs with cumulatively results for 337 patients were included (15-18). There is no evidence in the form of SNOT-22 or objective endoscopic score supporting postoperative nasal debridement following ESS. Although four of the six studies demonstrated some improvement in symptom score, only one of them was indicative of such an enhancement in the long term. However, a more frequent debridement regimen offers little improvement compared to debriding once or twice postoperatively. None of the reviewed studies evaluated the CRSwNP as a different subset of CRS from CRSsNP. Bugten et al. subgroup analysis showed that patients with CRSwNP seem to benefit the most from frequent debridement (19). 

Alsafar et al. found that debridement does not affect the overall quality of life and endoscopic scores; on the other hand, it negatively influences patient satisfaction and is associated with greater inconvenience (20). Varsak et al. found that frequent debridement causes more discomfort and facial pain and has more negative effects on work (21). Shi et al. reported that the benefit of frequent postoperative debridement was not in correlation with patients recovering from ESS (22). According to our review of literature, this study was the first RCT that assessed the short-term and long-term effects of debridement versus no debridement in CRSwNP and CRSsNP patients. Our analysis at 8 weeks postoperatively showed that in patients with CRSwNP, the debridement group had significant improvement in nasal blockage, sense of smell, SNOT-22 score, and Lund-Kennedy score. In patients with CRSsNP, the debridement not only did not result in the improvement of the SNOT-22 score but also aggravated the patients' quality of life at 8 weeks, although it was not significant. In CRSsNP patients, the debridement and control groups showed no significant difference in terms of patients' symptoms and Lund-Kennedy score. In contrast to observed benefits of debridement in CRSwNP at 8 weeks, no significant improvement was observed in the debridement group at 6 months in terms of VAS, SNOT-22, and Lund-Kennedy scores in CRSwNP and CRSsNP patients. 

## Conclusion

Based on the results of this study, debridement could lead to short-term improvements in CRSwNP patients; however, no long-term benefit was observed. Therefore, considering the healthcare costs and the required time and resources, postoperative debridement is not justified. Nonetheless, further studies are recommended to be performed to evaluate the consistency of our obtained result. 
